# Socioeconomic differences in psychiatric treatment before and after self-harm: an observational study of 4,280 adolescents and young adults

**DOI:** 10.1186/s12888-021-03654-9

**Published:** 2022-01-05

**Authors:** Joonas Pitkänen, Hanna Remes, Mikko Aaltonen, Pekka Martikainen

**Affiliations:** 1 Population Research Unit, Faculty of Social Sciences, University of Helsinki, P.O. Box 18, FIN-00014 Helsinki, Finland; 2 International Max Planck Research School for Population, Health and Data Science, Rostock, Germany; 3 Institute of Criminology and Legal Policy, Faculty of Social Sciences, University of Helsinki, Helsinki, Finland; 4 Law School, University of Eastern Finland, Joensuu, Finland; 5 Max Planck Institute for Demographic Research, Rostock, Germany; 6 Department of Public Health Sciences, Stockholm University, Stockholm, Sweden

**Keywords:** Self-harm, Young adulthood, Adolescence, Psychiatric treatment, Psychotropic medication, Socioeconomic differences, Parental education

## Abstract

**Background:**

Individuals in higher socioeconomic positions tend to utilise more mental health care, especially specialist services, than those in lower positions. Whether these disparities in treatment exist among adolescents and young adults who self-harm is currently unknown.

**Methods:**

The study is based on Finnish administrative register data on all individuals born 1986–1994. Adolescents and young adults with an episode of self-harm treated in specialised healthcare at ages 16–21 in 2002–2015 (n=4280, 64% female) were identified and followed 2 years before and after the episode. Probabilities of specialised psychiatric inpatient admissions and outpatient visits and purchases of psychotropic medication at different time points relative to self-harm were estimated using generalised estimation equations, multinomial models and cumulative averages. Socioeconomic differences were assessed based on parental education, controlling for income.

**Results:**

An educational gradient in specialised treatment and prescription medication was observed, with the highest probabilities of treatment among the adolescents and young adults with the highest educated parents and lowest probabilities among those whose parents had basic education. These differences emerged mostly after self-harm. The probability to not receive any treatment, either in specialised healthcare or psychotropic medication, was highest among youth whose parents had a basic level of education (before self-harm 0.39, 95% CI 0.34–0.43, and after 0.29, 95% CI 0.25–0.33 after) and lowest among youth with higher tertiary educated parents (before self-harm: 0.22, 95% CI 0.18–0.26, and after 0.18, 95% CI 0.14–0.22). The largest differences were observed in inpatient care.

**Conclusions:**

The results suggest that specialised psychiatric care and psychotropic medication use are common among youth who self-harm, but a considerable proportion have no prior or subsequent specialised treatment. The children of parents with lower levels of education are likely to benefit from additional support in initiating and adhering to treatment after an episode of self-harm. Further research on the mechanisms underlying the educational gradient in psychiatric treatment is needed.

**Supplementary Information:**

The online version contains supplementary material available at 10.1186/s12888-021-03654-9.

## Introduction

Estimates from psychological autopsy studies indicate that around 90% of suicide victims have a history of psychiatric disorders [1], and a similar prevalence (80%) has been shown in studies focusing on hospital-presenting non-lethal self-harm [2]. Moreover, around two-thirds of suicide-attempting adolescents have received mental health treatment before the attempt [3], and similar lifetime treatment histories have been found among adolescent [4] and adult [5] suicide victims. Hence, patient contact in healthcare services in general and mental healthcare in particular may provide opportunities for prevention of self-harm [6]. Furthermore, a hospital-presenting episode of self-harm might be an opportunity to establish patient contact and thus treat the underlying conditions [7].

However, not all people use healthcare equally. Previous research has documented socioeconomic differences by education, income and occupation in healthcare utilisation across different national contexts, healthcare systems and types of services [8–11]. A common finding in these studies is that there are negligible differences in general practitioner visits, but individuals in higher socioeconomic positions tend to use more specialist services [8, 10]. Besides differences in specialised service use, a recent study in Finland showed that individuals in lower socioeconomic positions are more likely to not use any healthcare [9]. In addition to healthcare in general, socioeconomic differences in service use have been documented in mental health care as well [12–16], and again especially in the use of specialised services. In addition to differences in plain economic resources, such as income, the observed differences in healthcare utilisation are likely to relate to differences in other socioeconomic resources as well, including, e.g., mental health literacy and knowledge of the mental healthcare system [17, 18].

Socioeconomic differences in mental health service use have been shown to exist among adolescents as well. Adolescents with lower levels of socioeconomic resources tend to have worse access to mental health treatment and use less services [17, 19], even though they often have more mental healthcare needs [20]. However, despite the fact that lower levels of socioeconomic resources are known to be associated with the risk of self-harm and mental ill health in adolescence and young adulthood [3, 6, 20], and socioeconomic resources have been shown to be associated with mental health treatment among suicidal adults [21], studies of socioeconomic differences in help-seeking or treatment utilisation among adolescents and young adults who self-harm are scarce. A review from 2012 identified only two such studies with inconclusive evidence [22], and we are unaware of any others. Instead of socioeconomic factors, previous research on help-seeking and treatment in this population has focused on, e.g., differences by age and sex, psychological factors, diagnosis of mental illness or use of medication [7, 22–24].

Documenting social disparities in treatment trajectories is important for increasing knowledge on health inequalities among youth who self-harm. In the current study, we aim to fill in this gap in research by employing longitudinal administrative register data on all Finnish children born in 1986–1994. We examine differences by parental education in the probabilities of specialised psychiatric care (inpatient admissions and outpatient visits) and psychotropic medication use before and after an episode of self-harm in adolescence or young adulthood. We focus on parental education, which captures differences in multiple areas of socioeconomic resources, including, e.g., economic resources, knowledge of healthcare systems and health literacy.

## Methods

### Data

The study is based on a longitudinal dataset (1986–2017) which contains administrative register data on all 0–14-year-old children living in Finland in 2000, linked with their biological parents. The data used in this study included sociodemographic information, including, e.g., age, sex, education level and income from Statistics Finland, prescription medication purchases from the Finnish Social Insurance Institution, and specialized healthcare service use from the Finnish Institute for Health and Welfare. The latter data includes all inpatient episodes and specialised outpatient visits at Finnish facilities providing hospital-level care, including emergency visits as well. Linkage between different data sources and children and their parents was done using anonymised identification numbers based on the Finnish personal identification system.

### Participants

From the entire available dataset, we limited our analyses to individuals born to Finnish parents between 1986 and 1994 and identified those who had a non-lethal episode of self-harm treated in specialised healthcare between age 16 and 21. Self-harm was conceptualised as any intentional self-injury or self-poisoning, irrespective of suicidal intent, and identified using information on external causes of injuries and poisonings (International Classification of Diseases Version 10 (ICD10) X69–X84; the Finnish classification codes all self-inflicted intentional poisonings as X69). Since we used healthcare data to identify self-harm, our study focuses only on severe cases, given that treatment in a hospital-level facility was required. Any self-harm at a community level is much more common than self-harm episodes treated in hospitals or other clinical services, and therefore the episodes involving treatment are sometimes referred to as the visible part of the iceberg [25, 26]. We limited the examined age range to 16–21 to achieve a study population that is comparable in terms of psychiatric treatment provided. First, in Finnish healthcare, there is a threshold between child psychiatry and youth psychiatry at age 13. Second, pharmacolocical treatment is less common in early adolescence [27, 28]. While severe self-harm does occur also in earlier ages, it is quite rare. For instance, in the data used in this study there were 444 children in the 1986–1994 birth cohorts who had self-harmed at age 14–15, and in our analytical sample, 71 (1%) had a previous episode of self-harm at these ages.

We defined the first case of self-harm between age 16 and 21 as the index episode, and as baseline year for covariate measurement the year 2 years prior to the year of the index episode. In total, there were 4,454 individuals who had an episode of self-harm between the defined age range, and who resided in mainland Finland at baseline. We excluded 20 individuals who emigrated, and 84 who died during the follow-up. Of the deaths, 52% were suicides and 34% accidental poisonings. We also excluded children with missing values of parental income at baseline (n*=*70). The final sample size was 4,280 individuals.

 We followed this sample for psychiatric treatment two years before and after the index episode. The earliest year of follow-up in the data was thus 2000 (children born 1986, with an index episode at age 16 and baseline year at age 14) and the latest 2017 (children born 1994, with an index episode at age 21 and baseline year at age 19). Of the 4,280 adolescents and young adults who had self-harmed between age 16 and 21, 64% were girls. 81% of the index episodes were due to self-poisoning, and the median age of self-harm was 19.

### Measures

#### Outcomes

Using the healthcare data provided by the Finnish Institute for Health and Welfare, monthly admissions into inpatient psychiatric care and outpatient visits to specialised psychiatric care (yes/no) two years before and after the episode of self-harm were defined with ICD10 codes F10–F16, F18–F69, and F80–F99. Psychotropic medication purchases were identified from the register of prescription medication purchases using the Anatomical Therapeutic Chemical Classification codes N05, N06A (excluding N06AD), and N07B (excluding N07BA) on a similar monthly basis.

#### Parental education

Parental highest education at the baseline year was obtained from Statistics Finland’s data. We derived educational information in the baseline year for both biological parents and used the highest education as our exposure variable. Education was classified into four different categories based on Statistics Finland’s classification, which is based on UNESCO’s International Standard Classification of Education 2011 (ISCED 2011) [29]. The following categories were used: basic (ISCED 0–2), upper secondary (ISCED 3–4), lower tertiary (ISCED 5–6) and higher tertiary (ISCED 7–8). In the case where parental education at baseline was missing, we used the latest available observation.

#### Covariates

We controlled our analyses for family income, a possible mechanism for differences in treatment by parental education. We used annual household consumption income deciles from Statistics Finland data, measured at baseline for those living with their parents. For those who had already left the parental home at baseline, information on income was derived from the latest year they lived with their parents.

As covariates, we included an indicator for age at index self-harm (below 18/18 or older) and year of index episode (continuous) to control for differences related to healthcare system (youth psychiatry vs. adult psychiatry) and secular increase in both specialised psychiatric treatment [30] and prescriptions of psychotropic medication [31]. We also included sex as a covariate and adjusted for university-hospital–specific catchment areas at baseline to account for possible area-level differences in treatment provision.

### Statistical modelling

#### Trajectories

We combined calendar time into three-month periods before and after the index self-harm, based on months of admission or visit. We excluded all the psychiatric inpatient admissions and outpatient visits that started within seven days from the date of admission or discharge of the self-harm episode to avoid falsely counting the self-harm episode into specialised psychiatric treatment before or after. For outcome symmetry and because the data does not include medication delivered at hospitals, we similarly excluded medication purchases within seven days of self-harm.

In our main analyses, we excluded the calendar month of the index episode for two reasons. First, it is likely that individuals receive a diagnosis during the reference episode of self-harm, which introduces a sharp peak to trajectories and hence may obscure more moderate changes in graphical presentation of results. Second, purchases of psychotropic medication are more likely to occur during a three-month period than during a much shorter one-month period, which introduces a spurious drop in the index month. We present a detailed description of the formation of trajectory data in Additional File 1 and an illustration of the time-related discordance in terms of observed means in Additional File 2. In calculating these observed means, all the inpatient admissions, outpatient visits and medication purchases, including those occurring within seven days of self-harm, were included.

To the pooled data containing 16 three-month periods for each individual, we fit a logistic Generalized Estimation Equations model with an unstructured correlation matrix. The model includes main effects of parental education, time relative to self-harm and their interaction term, which allows for separate modelling of treatment trajectories by parental education. We present the trajectories by parental education as average predicted probabilities of treatment at each three-month interval.

#### Cumulative treatment

To assess the cumulative treatment probability and potential differences in types of treatment, we also pooled treatment into six periods: ever (during the two years), one year, and one month before and after self-harm. For each of these periods, we combined the hospital data with the medication data and created a treatment outcome with four categories of no treatment, medication only, outpatient treatment and inpatient treatment. Those in the inpatient category could have also received outpatient treatment. Exact dates relative to the index episode of self-harm were used to determine the outcomes at each period, and outcome events occurring within seven days of self-harm were again excluded. We modelled these outcomes with multinomial regression using parental education as the exposure variable. In the models, standard errors were clustered by maternal ID to account for correlation between siblings in data. All the models were adjusted for all the covariates.

Finally, we investigated the cumulative probability of treatment across the whole follow-up period, calculated as the cumulative average value of ever receiving treatment at every three-month interval. In these analyses, we also included the index month and the events occurring within seven days of self-harm to fully assess when the individuals receive their first psychiatric diagnosis or medication prescription relative to self-harm.

## Results

### Descriptive statistics

Table 1 shows the cumulative prevalence of the outcome variables one year before and after self-harm and the distributions of the baseline covariates by parental education. To provide further insights into the characteristics of the study population, we present descriptive statistics for the birth cohorts 1986–1994 both among the adolescents and young adults who self-harmed and in the general population in 2009 (the middle year of our follow-up) in Additional File 3.

Based on Table 1, there were only small differences in the methods of self-harm between the parental education groups. Adolescents and young adults who self-harmed were more commonly males in the lowest two parental education groups, while females were over-represented in the two highest parental education groups. Age at self-harm did not differ to a great extent by parental education. The share of under 18-year-olds was 5% points larger among the adolescents and young adults whose parents had a higher tertiary education than the same share among those whose parents only had basic level of education.

There was a clear educational gradient in both specialised psychiatric care and psychotropic medication (Table 1). Children whose parents had the highest education had the most outpatient visits, inpatient admissions and psychotropic medication purchases both before and after self-harm, whereas those whose parents had the lowest level of education had the lowest prevalence of any of these treatments. The prevalence of different types of treatment in the middle educational groups fell in between these two extremes.

**Table 1:** Distributions of baseline covariates and psychiatric treatment one year before and after self-harm by parental education, among adolescents and young adults who self-harmed at ages 16–21 in 2002–2015 (n= 4,280)

	Parental education							
	Basic		Upper secondary		Lower tertiary		Higher tertiary	
	N	%	N	%	N	%	N	%
*Method of self-harm*								
Poisoning	373	80	1,696	80	1,027	82	372	82
Other	93	20	418	20	222	18	79	18
*Specialised psychiatric care one year before self-harm*								
No	292	63	1,254	59	711	57	226	50
Outpatient only	98	21	473	22	276	22	115	26
Inpatient	76	16	387	18	262	21	110	24
*Specialised psychiatric care one year after self-harm*								
No	245	53	1,009	48	530	42	160	35
Outpatient only	135	29	627	30	417	33	171	38
Inpatient	86	18	478	23	302	24	120	27
*Psychotropic medication purchases one year before self-harm*								
No	250	54	1,022	48	596	48	189	42
Yes	216	46	1,092	52	653	52	262	58
*Psychotropic medication purchases one year after self-harm*								
No	232	50	910	43	456	37	149	33
Yes	234	50	1,204	57	793	63	302	67
*Income decile*								
Lowest five deciles	409	88	1,649	78	689	55	142	31
Highest five deciles	57	12	465	22	560	45	309	69
*Sex*								
Male	198	42	813	38	411	33	140	31
Female	268	58	1,301	62	838	67	311	69
*Age at self-harm*								
14–17	91	20	402	19	274	22	112	25
18–21	375	80	1,712	81	975	78	339	75

### Treatment trajectories

#### Predicted trajectories of inpatient admissions and outpatient visits

**Fig. 1:**
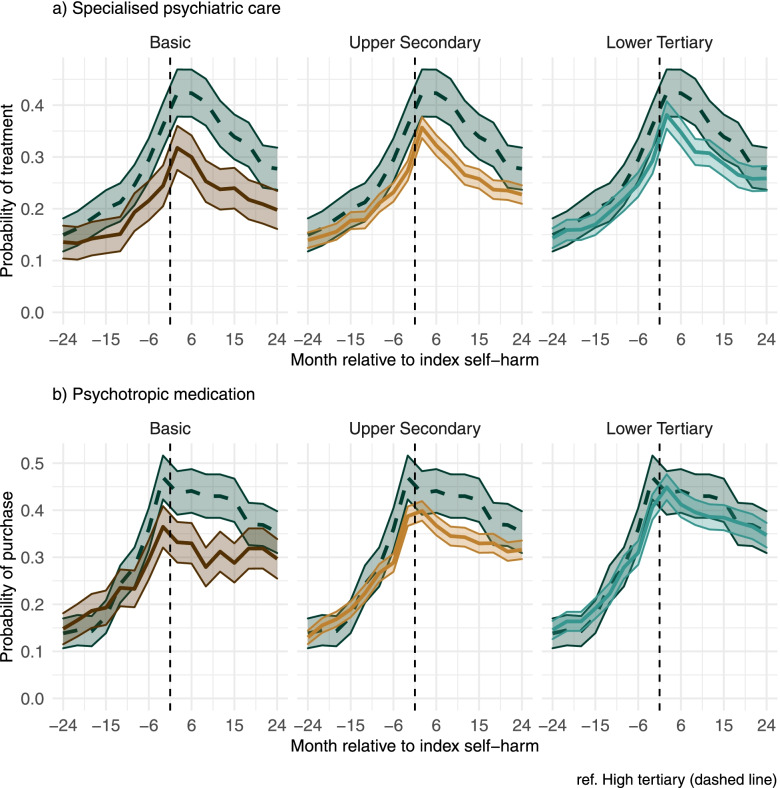
Predicted probabilities of (**a**) specialised psychiatric care and (**b**) psychotropic medication purchases by parental education. The dashed line indicates higher tertiary education and is used as the reference line in each plot. Values on x-axis refer to the three-month periods which include the denoted months

We present the predicted probabilities of specialised psychiatric care and medication purchases before and after self-harm by parental education in Fig. 1. To illustrate the uncertainty of the estimates with 95% confidence intervals, we use higher tertiary education as a reference (dashed line) and compare the other groups to that one at a time. The observed means of psychiatric treatment including the month of self-harm are shown separately by sex in Additional File 2.

The probability of psychiatric inpatient or outpatient treatment at the start of follow-up, 24–22﻿ months before self-harm, was similar in all the groups of parental education, ranging between 0.14 and 0.15. In all the groups, the probability increased when nearing the index self-harm episode, and at 1–3 months prior to self-harm, the probabilities ranged between 0.24 (95% CI: 0.20, 0.28) among the individuals whose parents had a basic level of education and 0.36 (0.32, 0.40) among the individuals in the group of parental higher tertiary education. Overall, the educational trajectories followed a gradient: the higher the parental education, the higher the probability of admission.

The probability of psychiatric inpatient or outpatient treatment peaked at 1–3 months after self-harm in all educational groups and started to decrease afterwards. The probabilities ranged between 0.32 (95% CI: 0.27, 0.36) among children of the parents with basic education and 0.42 (0.38, 0.47) among children of the parents with the highest education level. After the peak, the gap between the lower two levels of education and highest level of education is clearly visible in Fig. 1, until the trajectories near each other in the end of the follow-up. All in all, parental lower tertiary education did not differ much from higher tertiary during the follow-up.

#### Predicted trajectories of psychotropic medication

The trajectories of psychotropic medication purchases by parental education were broadly similar to the predicted trajectories of specialised psychiatric care. At 1–3 months before self-harm, the probabilities ranged between 0.36 (0.32, 0.41) in the group of parental basic education and 0.47 (0.42, 0.52) in the group of parental higher tertiary education. These were also the peaks of medication purchases in these two groups across the whole follow-up. In contrast to the ends of the educational distribution, the two intermediary groups had the highest probability of purchases 1–3 months after self-harm. Consistent with the pattern in specialised psychiatric care, the probability of purchases started to decrease when moving forwards from self-harm. The educational differences after self-harm resembled those in specialised psychiatric care: the higher the education, the higher the probability of medication purchase, but the differences diminished near the end of the follow-up period. Lower tertiary education did not differ from higher tertiary at any point of the follow-up.

### Pooled outcomes and cumulative trajectories

**Fig. 2:**
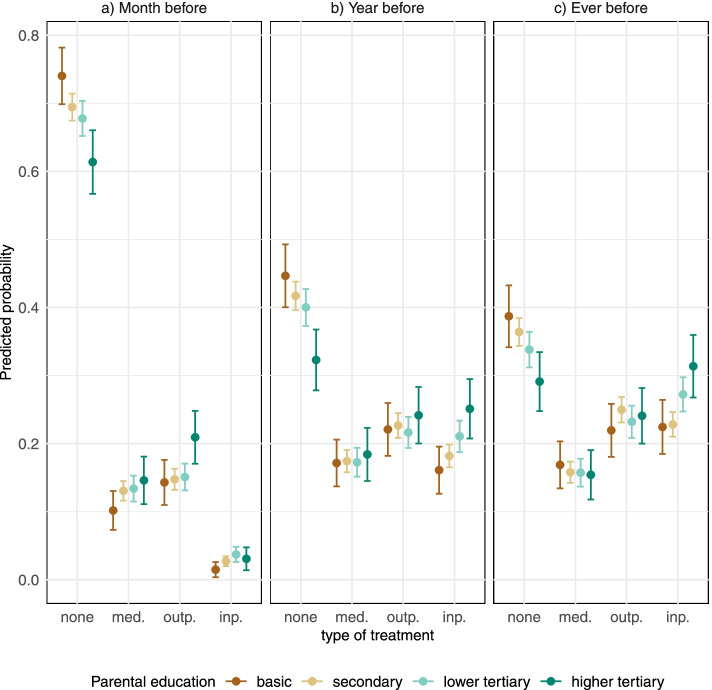
Predicted probabilities and 95% confidence intervals of type of treatment (no treatment, medication only, outpatient treatment, and inpatient treatment) before self-harm. Results from multinomial logistic regression models

**Fig. 3:**
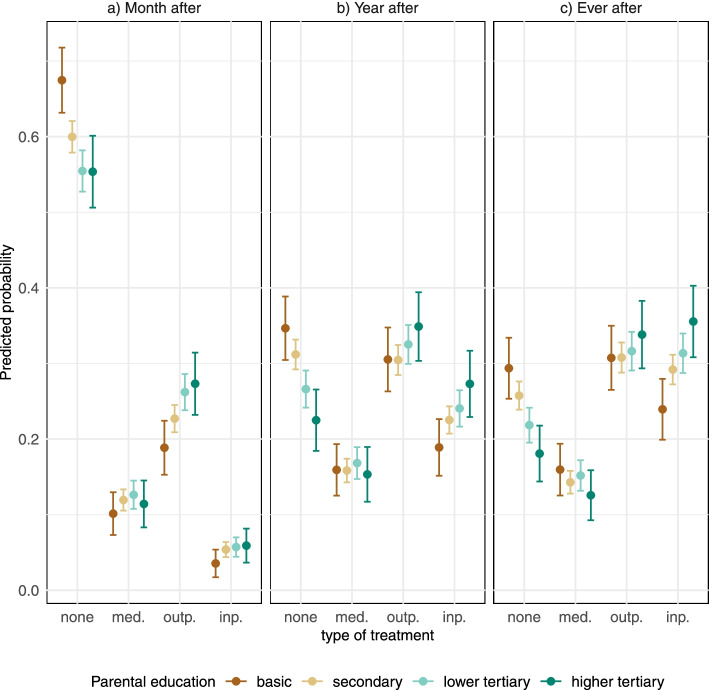
Predicted probabilities and 95% confidence intervals of type of treatment (no treatment, medication only, outpatient treatment, and inpatient treatment) after self-harm. Results from multinomial logistic regression models

In the treatment-type analyses, the outcome variable consisted of four categories, which were determined by all the inpatient admissions, outpatient visits and medication purchases pooled over a specific time range. The categories were no treatment, only medication, outpatient treatment, and inpatient treatment. The outcome was analysed with multinomial models. Overall, the results from treatment-type analyses confirm the results from the trajectory models, but some new insights also emerged (Figs. 2 and 3).

The clearest educational gradients were in the probabilities of not receiving any treatment (Fig. 2): at all the time points, children of parents with basic education had the highest probability of no treatment (probability ever before 0.39, 95% CI 0.34–0.43), whereas adolescents and young adults whose parents had higher tertiary education stood out as least likely to not receive any treatment (ever before 0.22, 95% CI 0.18–0.26).

The findings on treatment after self-harm (Fig. 3) were similar: probability of not receiving any treatment was the highest among the children of the parents with basic education (ever after 0.29, 95% CI 0.25–0.33) and lowest among the group of parental higher tertiary education (ever after 0.18, 95% CI 0.14–0.22).

Comparable differences were found in inpatient treatment, with the highest probabilities of this outcome one and two years before and after self-harm among children of higher tertiary educated parents and lowest among children of basic educated parents. In contrast, the educational differences were negligible in the probability of outpatient treatment one or two years before self-harm, but there was an educational gradient in specialised outpatient visits a month before and after self-harm (Figs. 2 and 3). The probability of receiving only medication did not differ by parental education across the whole follow-up.

**Fig. 4:**
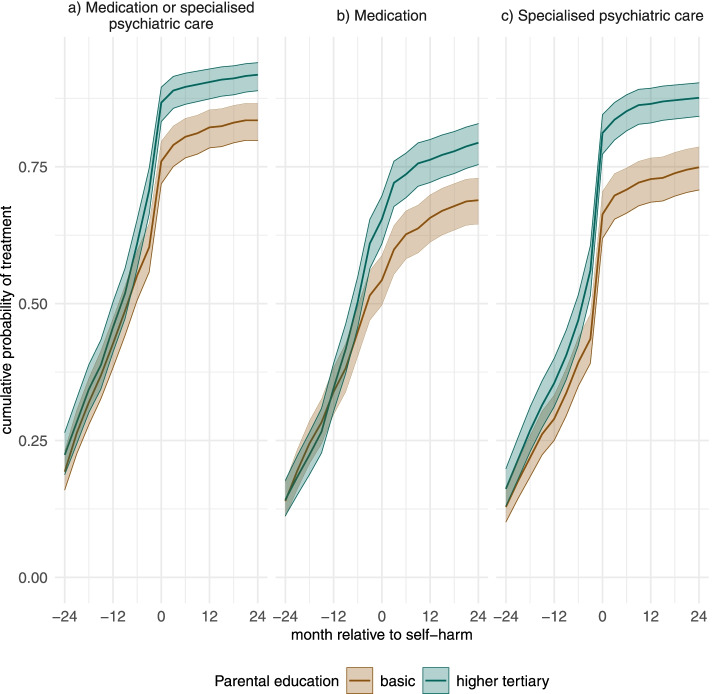
Cumulative observed averages of experiencing (**a**) either treatment, (**b**) psychotropic medication or (**c**) specialized psychiatric care in the groups of parental higher tertiary education and parental basic education. ﻿Values on x-axis refer to the three-month periods which include the denoted months

Cumulative probabilities in Fig. 4 confirm that most of the differences between the ends of the educational distribution are due to differences of not receiving treatment after self-harm, especially specialised mental healthcare. The cumulative means of treatment in these two educational groups started to diverge from each other in the months preceding self-harm and were clearly different thereafter. At the end of the follow-up, the cumulative average of either treatment among higher tertiary educated parents was 0.92 (95% CI 0.89–0.94) and among the lowest educated parents 0.83 (95% CI 0.80–0.87).

## Discussion

The results in this study show differences by parental education in specialised psychiatric care and psychotropic medication use among youth who self-harm. We observed divergence in treatment mostly after an episode of self-harm and the most pronounced differences by parental education between the highest and lowest educational groups. In addition, our results suggest that the clearest differences emerge when examining specialised healthcare use, which was clearly more common among children of higher educated parents than their peers with the lowest educated parents. Although similar results regarding socioeconomic differences have been previously found among adults who self-harm [21], to our knowledge, this is the first study to present disparities in treatment among adolescents and young adults, in specific time periods both before and after an episode of self-harm, and by different types of treatment.

The vast majority of the study sample received some mental health treatment during the follow-up, but there was a persistent difference by parental education in not receiving any treatment over the four-year follow-up period. Since the differences were less pronounced before self-harm, these findings indicate that individuals with less educated parents are more prone to discontinue specialised psychiatric care or medication use or they do not start treatment at all after an episode of self-harm. Since the observed trajectories were relatively similar in shape, it seems likely that not starting treatment explains most of these findings. Moreover, differences by parental education were visible in treatment during the index month of self-harm as well, suggesting that youth with lower educated parents do not receive mental health diagnoses when presenting to specialised healthcare due to self-harm in a similar manner as their peers with higher educated parents. In treatment-type analyses, children of higher tertiary educated parents were observed to have a higher probability of inpatient treatment, whereas differences in other treatment types were smaller.

The observed differences by parental education in treatment-seeking and service use are likely to relate to differences in parental resources. A plausible candidate would be economic resources. However, pure material resources are not likely to explain the educational disparities in treatment in this study: the data used in the study includes publicly funded specialised healthcare and medication use, which is reimbursed to a great extent, and we additionally controlled for income at baseline. Therefore, levels of non-material resources, such as knowledge related to mental ill-health and treatment, social networks, and will and ability to demand services [11, 17], which often are at a higher level among higher educated parents [32], could thus explain these differences. Furthermore, children with higher educated parents might have higher levels of trust and fewer conceptions of stigma related to mental health treatment and thus they might be more compliant with starting treatment [11, 18]. Finally, educational background may influence communication between parents, patients and healthcare professionals, and the level of information received by the parents might also differ by their education [33]. Unfortunately, with our data, we could not assess whether these observed disparities emerged from deliberate decisions to not establish a treatment connection, or from structural factors related to access to care. Further research on these mechanisms is needed, as it seems evident that youth whose parents have lower levels of education might need more support in accessing, commencing and adhering to mental health treatment. This should be an international effort; although it is likely that similar disparities would be observed in different contexts, it is plausible that the underlying mechanisms differ according to the contextual properties of healthcare systems and other contextual social factors.

### Methodological considerations

A limitation of the study is that we did not have access to data from primary healthcare. A common finding in healthcare service use literature is that higher levels of socioeconomic resources increase the use of specialised services but individuals in lower positions use equally or more general healthcare [8, 9, 11, 12, 21]. In addition, we did not have access to information on private services, more commonly used among individuals with more resources. However, we also used data on psychotropic medication purchases, on which we have data whether prescribed in public or private healthcare. Since we also observed educational differences (although somewhat less pronounced) with this outcome, our findings on the educational differences seem robust. In addition, even though the initial contacts regarding mental health problems in the Finnish context may occur in primary or private healthcare, in the cases where these problems are deemed severe enough or the offered treatment does not suffice, the doctors refer the more severe cases to publicly provisioned specialised healthcare. Hence, our data should capture the individuals who are treated for severe mental health problems quite well.

A second limitation also concerns the use of healthcare data. Previous work has encountered possible misreporting and incomplete data in several hospital districts in the specialised outpatient health care dataset [30, 34]. We repeated our main analyses excluding hospital districts that seemed to have incomplete data, but the results were similar, and we decided to use all data available. Overall, incomplete data does not seem to bias our results.

The main strength of this study lies in the population-representative data and the high level of accuracy in recording the timing of the events in data as well as the ability to link different types of treatments together. We were able to use daily recorded objective measures of clinical diagnoses and prescription medication on all the individuals among all the members of 9 birth cohorts who self-harmed at ages 16–21. Although self-harm treated in specialised healthcare is relatively uncommon in the population, thanks to the sample size we were able to identify over 4000 youth with a clinically recorded episode of self-harm. Moreover, we could accurately link parental education to their offspring. Finally, a further advantage of using register data is there are not challenges related to non-response, attrition during follow-up or recall bias common in studies based on surveys and patient populations.

### Implications for future research

Besides the mechanisms generating the observed socioeconomic differences, several further research questions emerge from the findings of the current study. First, in this paper, all different psychiatric diagnoses and classes of psychotropic medication were used together to assess socioeconomic differences in psychiatric treatment in general. However, specific diagnoses may have unique impacts on the risk of self-harm [35]. The prevalence of certain psychiatric disorders is larger than the prevalence of others [2], and comorbidities [15] and symptom severity [36, 37] might influence treatment-seeking behaviours. Whether these more specific characteristics of psychiatric morbidity interact with socioeconomic resources in treatment-seeking among adolescents and young adults who self-harm is a question that remains open after this study. Previous research does suggest that adolescents and young adults with low levels of parental education might have more severe symptoms [38], but, on the other hand, symptom severity might also eradicate socioeconomic differences in treatment utilisation [36].

Besides a closer inspection of the underlying disorders, further research should delve deeper into socioeconomic differences in treatment quality and the type of treatment received. In the Finnish context, psychiatric treatment is primarily voluntary, but under certain conditions, including suicidal behaviour, patients may be involuntarily placed into care or be subject to coercive measures (e.g., restraint and seclusion) [39–43]. To our knowledge, socioeconomic differences in involuntary placements or coercive measures have not been examined.

Finally, to avoid loss of statistical power due to low cell counts when using a four-category variable for parental education, we used sex as a covariate rather than a stratifying variable. However, it is known from previous literature that women more often engage in non-lethal self-harming behaviours [26], and are also more likely to seek treatment than men [23, 24]. Therefore, it would be of interest for future research to formally study whether sex modifies the observed treatment trajectories. The investigation of sex differences could be extended to cover differences between mothers and fathers and their socioeconomic resources as well.

### Clinical and policy implications

The clinical and policy implications derived from these results need to be presented in a speculative manner as we were not able to identify the mechanism producing the disparities. If the adolescents and young adults who self-harm do not receive treatment because they do not know where to access it, they do not trust the practitioners or are afraid of economic costs or stigma associated with being in mental healthcare, the differences by educational background might be attenuated by increasing the general knowledge on the mental healthcare system and mental health literacy among both parents and their children of all educational backgrounds. The educational system might play a key role here, and various universal mental health promotion programmes with community and family involvement have been implemented in different national contexts [44]. However, the effectiveness of these interventions remains largely an open question [44].

Reasons for not seeking help might also relate to the relationship between the healthcare professionals and patients [17, 45]. Previous literature indicates that in doctor–patient communication, doctors provide less support and information for individuals in lower socioeconomic positions [33], and there is also evidence that implicit biases related to socioeconomic and demographic factors might influence the interaction between healthcare professionals and patients [46]. Increasing the awareness of these communicative differences and attitudes among healthcare professionals could improve their interaction and relationships with the patient [33], which could motivate self-harming patients to better enter and adhere to treatment. In particular, further training of primary healthcare professionals, who are the first contacts between the patient and mental healthcare, may prove to be beneficial.

However, if the observed socioeconomic disparities relate to structural barriers in access inherent to the healthcare system, the implications are different. The Finnish healthcare system is based on the principles of universal healthcare promotion and equal access to services. Despite that, the Finnish system has comparatively high levels of inequality in need-adjusted service use [47]. These inequalities have been at least partially attributed to occupational and private healthcare [8, 9], to which socioeconomically disadvantaged individuals have more limited access. Additional structural barriers emerge, for instance, if the existing services are not available due to distance, crowding or long waiting times, or if the services have out-of-pocket costs [9, 17, 47, 48]. Currently a restructuring of Finnish health and social services is underway, which aims to reduce the inequality in access to healthcare related to these structural barriers [49]. Whether the restructuring will attenuate the disparities in psychiatric care observed in this study, remains to be seen.

## Conclusions

The findings from this study demonstrate that around half of youth who self-harm have been either admitted to specialised psychiatric care as an inpatient or outpatient, or used psychotropic medication during the year before self-harm, but also that there is a considerable proportion of individuals who do not receive any type of specialised treatment or psychotropic medication before or after self-harm. Importantly, lower parental education is associated with lower levels of specialised psychiatric inpatient admissions and outpatient visits and medication use especially after self-harm. In general, strategies to support first contacts with specialised mental health care, treatment continuity and adherence to treatment is likely to be beneficial for self-harm prevention and treatment of severe underlying psychiatric disorders, in particular among children from families with lower levels of socioeconomic resources. Further research on the possible mechanisms behind help-seeking, access to treatment and the observed differences by parental education are needed to determine suitable modes of support.

## Supplementary Information

**Additional file 1.**

**Additional file 2.**

**Additional file 3.**

## Data Availability

The data that support the findings of this study are available from FinData (https://www.findata.fi/) but restrictions apply to the availability of these data, which were used under license for the current study, and so are not publicly available. Data are however available from the authors upon reasonable request and with permission of FinData.

## Abbreviations

ATC: Anatomical Therapeutic Chemical Classification System
ICD10: International Classification of Diseases Version 10
