# Supplementary material for: Socioeconomic differences in psychiatric treatment before and after self-harm: an observational study of 4,280 adolescents and young adults
Source: BMC Psychiatry. 2022 Jan 5;22:14. doi: 10.1186/s12888-021-03654-9 (PMC8728977; doi:10.1186/s12888-021-03654-9)
Supplement: Supplementary file 1 — Additional file 1. [file 12888_2021_3654_MOESM1_ESM.docx]

**Additional file 1: Formation of trajectory data**

The hospital data used in this study is relatively complex as the dataset includes all the hospital-level inpatient and outpatient admissions and there are multiple overlapping and nested episodes (Figure 1). These episodes need to be combined into separate episodes with at least one-day difference between the date discharge of the first episode and date of admission of the second. We started the procedure by identifying all the self-harm and psychiatric care episodes, based on diagnostic information and excluded all the other episodes. Second, we identified nested episodes. These episodes had later date of admission than previous episode but they ended at the same time or earlier as the first episode. We copied the information of psychiatric treatment and self-harm, and information on inpatient and outpatient (emergency room visits included) treatment from these nested episodes, after which we excluded the redundant nested admissions.


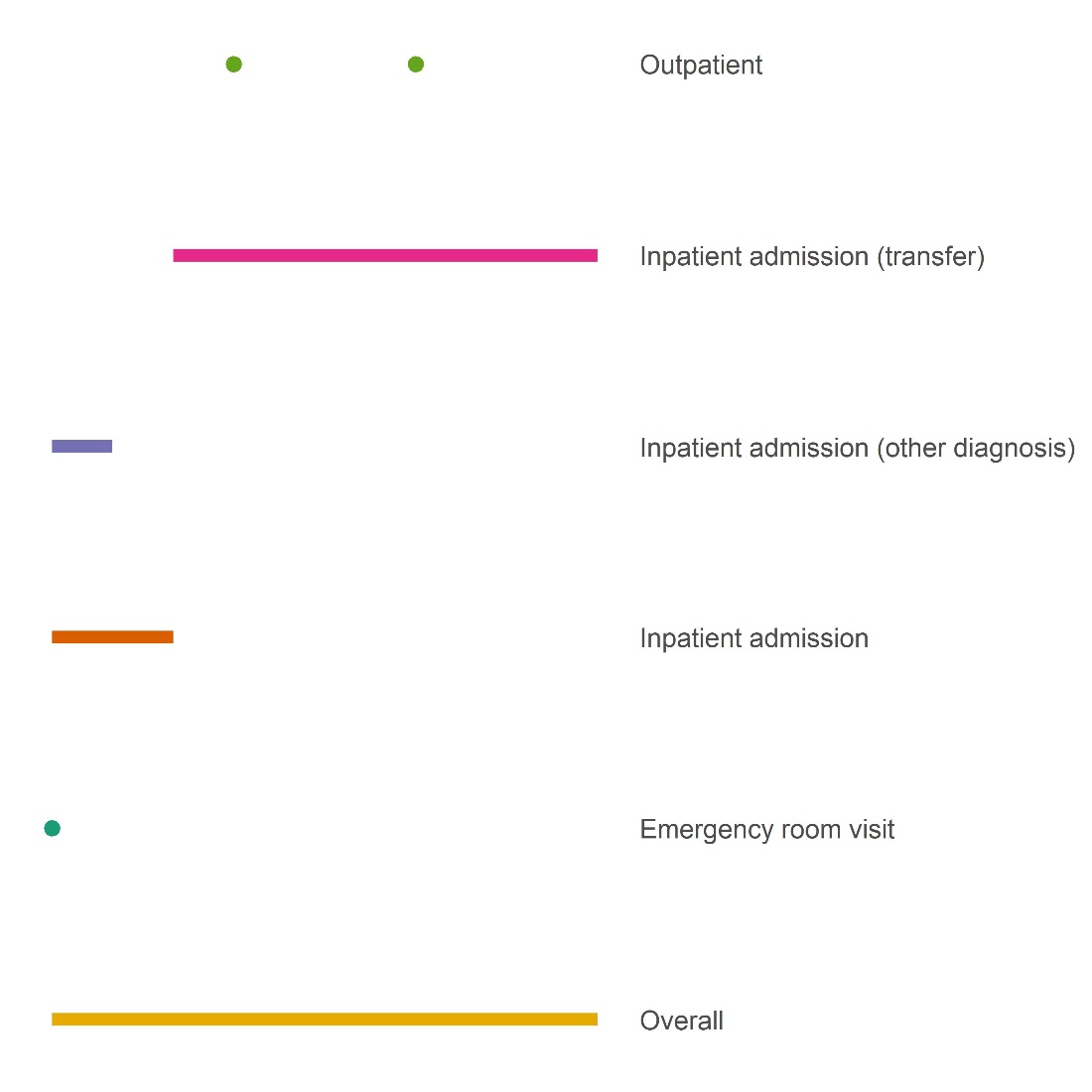


**Figure A1.1: Example of a hypothetical episode consisting of multiple episodes in the hospital data**

Second, we identified treatment chains and overlapping episodes. If an episode started at the same date as the previous ended, or started during the earlier episode but ended later, we considered these as chained or overlapped episodes. From these episodes, we took the latest date of discharge and used it as the end date of the first episode. We copied information from all the consequent and overlapping information to the first episode and removed the redundant data. As the result, we had only separate episodes including all the relevant data from the nested, consequent and overlapping episodes (The Overall line in Figure 1).

From this modified dataset, we first identified the self-harm episodes used as the time index for psychiatric treatment trajectories and then the monthly psychiatric hospital-level care. We used information on the month of admissions and pooled all the psychiatric episodes together by month and year. If the psychiatric episode had started in seven days before the index date of self-harm, or in seven days after the discharge date of self-harm, the episode was not included. Lastly, we limited the follow-up to cover two years before and after the index self-harm and pooled monthly treatment into three-month periods. The resulting final dataset used in analyses thus includes 17 rows per individual, of which eight three-month periods before and after the index month and a one-month period at index month. The latter was not used in analyses. Table 1 illustrates a hypothetical individual with an index self-harm episode starting 2 July at some arbitrary year.

| **Table A1.1: Data structure for a hypothetical individual** | | |
| --- | --- | --- |
| Date range | Time to index | Treatment |
| 01/07-30/09 | -24-22 | 0 |
| 01/10-31/12 | -21-19 | 0 |
| 01/01-31/03 | -18-16 | 0 |
| 01/04-30/06 | -15-13 | 1 |
| 01/07-30/09 | -12-10 | 0 |
| 01/10-31/12 | -9-7 | 1 |
| 01/01-31/03 | -6-4 | 1 |
| 01/04-30/06 | -3-1 | 0 |
| 01/07-31/07 | Index | NA |
| 01/08-31/10 | 1-3 | 1 |
| 01/11-31/01 | 4-6 | 1 |
| 01/02-30/04 | 7-9 | 0 |
| 01/05-31/07 | 10-12 | 0 |
| 01/08-31/10 | 13-15 | 0 |
| 01/09-31/01 | 16-18 | 1 |
| 01/02-30/04 | 19-21 | 0 |
| 01/05-31/07 | 22-24 | 0 |
