# Supplementary material for: Socioeconomic differences in psychiatric treatment before and after self-harm: an observational study of 4,280 adolescents and young adults
Source: BMC Psychiatry. 2022 Jan 5;22:14. doi: 10.1186/s12888-021-03654-9 (PMC8728977; doi:10.1186/s12888-021-03654-9)

**Additional file 2: Observed means of specialised psychiatric admissions and psychotropic medication purchases by time point before and after self-harm**


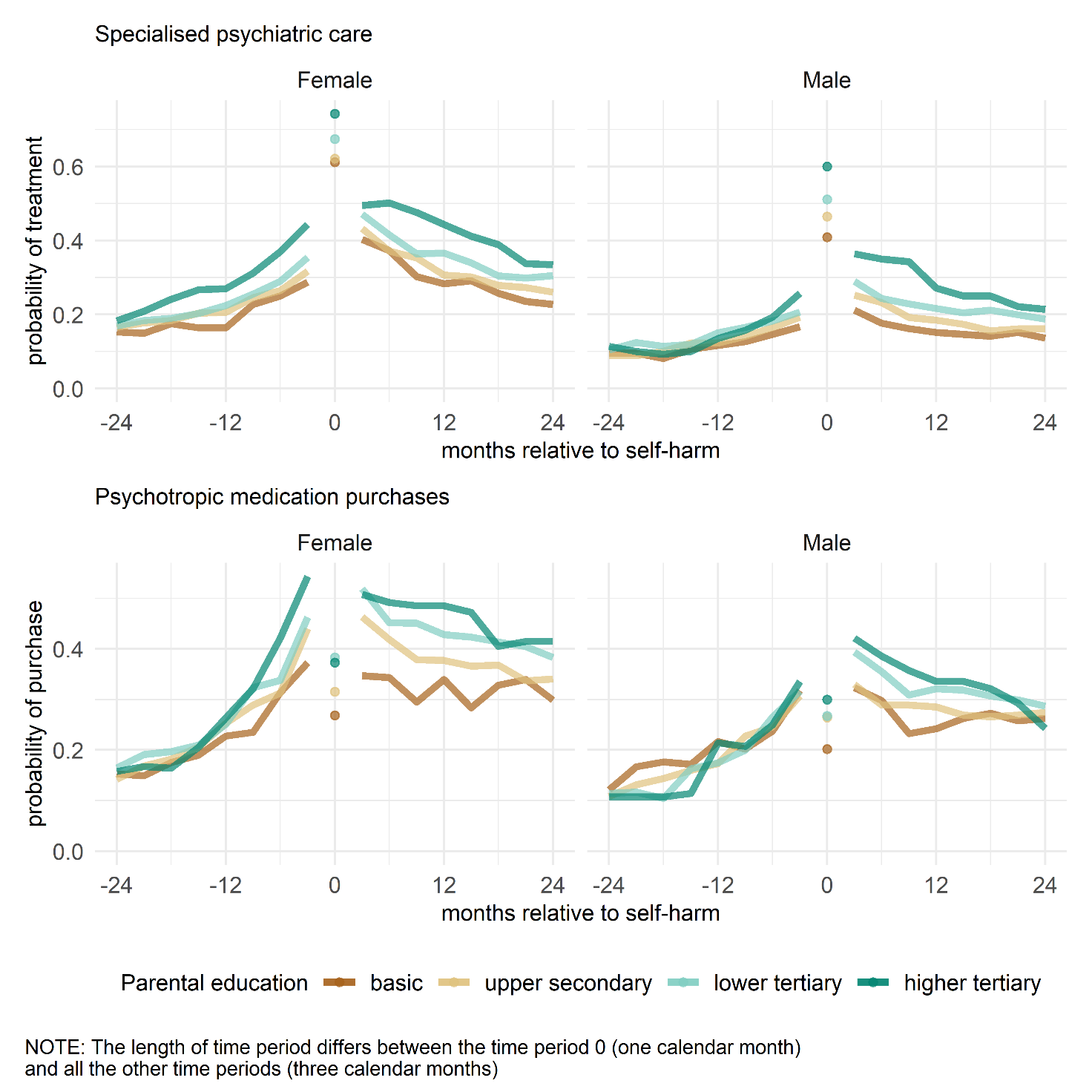

Supplement: Supplementary file 2 — Additional file 2. [file 12888_2021_3654_MOESM2_ESM.docx]
