# Supplementary material for: Socioeconomic differences in psychiatric treatment before and after self-harm: an observational study of 4,280 adolescents and young adults
Source: BMC Psychiatry. 2022 Jan 5;22:14. doi: 10.1186/s12888-021-03654-9 (PMC8728977; doi:10.1186/s12888-021-03654-9)
Supplement: Supplementary file 3 — Additional file 3. [file 12888_2021_3654_MOESM3_ESM.docx]

**Additional File 3: Comparisons between the population who self-harmed and general population aged 15-23 in 2009**

| Table 1: Distributions of socioeconomic variables and psychiatric treatment among youth who self-harmed and general population | | |
| --- | --- | --- |
|  | Youth who self-harmed^c^ | General population in 2009^d^ |
| **Specialised psychiatric treatment^a^** | N (%) | N (%) |
| No | 2483 (58) | 533594 (96) |
| Outpatient only | 962 (22) | 16509 (3) |
| Inpatient | 835 (20) | 4113 (1) |
| **Psychotropic medication^b^** |  |  |
| No | 2057 (48) | 521902 (94) |
| Yes | 2223 (52) | 32314 (6) |
| **Parental education** |  |  |
| Basic | 466 (11) | 29643 (5) |
| Upper secondary | 2114 (49) | 232579 (42) |
| Lower tertiary | 1249 (29) | 202364 (37) |
| Higher tertiary | 451 (11) | 89630 (16) |
| **Income** |  |  |
| In the lowest five deciles | 2889 (68) | 277792 (50) |
| **Total** | 4280 | 554216 |
| Only individuals with Finnish background included | | |
| ^a,b^Specialised treatment and medication measured the year before self-harm (excluding week before), or in 2009 for the general population sample.  ^c^Individuals born 1986–1994 with an episode of self-harm at ages 16–21  ^d^General population in 2009 aged 15–23, born 1986–1994 | | |

For comparative purposes, we derived information on parental education and household income from Statistics Finland’s data for all the children born 1986–1994 to Finnish-born parents and residing in mainland Finland in 2009, excluding children with missing data. We linked all the psychotropic medication purchases from the Finnish Social Security Institute’s register, and psychiatric inpatient admissions and outpatient visits from the Finnish Institute for Health and Welfare’s Care register for Health Care with the sociodemographic data.

Compared to the general population, individuals who self-harmed had less educated parents and lower family income. Among youth who had self-harmed, both psychiatric admissions and visits, and purchases of psychotropic medication the year before the year of self-harm were highly common, as around 40% and 50% of them had a record of admission/visit or medication, respectively. These figures were 8–10 times higher than the general population prevalence.
